# Interventions for spinal muscular atrophy: an impetus for newborn screening

**DOI:** 10.1016/j.ebiom.2021.103507

**Published:** 2021-07-24

At the end of May, 2021 a 5-month-old baby became the first in the UK to receive Zolgensma, a gene replacement therapy for spinal muscular atrophy (SMA). His treatment follows a deal announced in March between the UK's National Health Service and Novartis that will see the £1•795 million drug sold for an undisclosed discount. Following approvals by regulatory bodies, including the US Food and Drug Administration (FDA), European Medicines Agency and Japanese Ministry of Health, Labour and Welfare, the life-changing therapy is now available in up to 40 countries worldwide. Novartis have reconfirmed their commitment to a global managed access programme that will see the allocation of 100 doses per year free-of-charge to children who would otherwise not have access to the treatment.

SMA is a group of disorders characterised by loss of motor neurons in the spinal cord, resulting in muscle atrophy, weakness, and paralysis. The progressive muscle wasting affects all voluntary muscles, leading to movement and respiratory problems. The severity of disease varies with age of onset. In the most severe form (type 1), symptom onset begins within the first 6 months of life and is associated with a typical life expectancy of less than 2 years. Less severe forms (types 2 and 3) are diagnosed later in childhood, while type 4 typically presents in adulthood.

SMA is an autosomal recessive condition caused by mutation in the *SMN1* gene, which encodes a protein (SMN) essential for motor neuron survival. 94% of cases have a deletion affecting exon 7 of *SMN1.* The phenotypic severity of SMA is modified by the presence of copy number alterations in a second gene, *SMN2*, that also encodes SMN. A single nucleotide difference between the two genes results in approximately 90% of the transcripts from *SMN2* producing a truncated, biologically inactive protein. Genetic testing can predict the clinical course of SMA and is important to inform medical care for the patient, which, until recent years, was mainly supportive.

Zolgensma was the second therapy to be approved globally for SMA treatment, receiving approval from the US FDA in 2019. It is a gene replacement therapy that delivers a functional copy of *SMN1* in an adeno-associated virus 9 vector. In May in JAMA Neurology, the 5-year interim results of the long-term extension of the START trial, reported the safety and clinical durability of onasemnogene abeparvovec (the generic name for Zolgensma). 13 children (median age 38•9 months) with type 1 SMA and 2 copies of *SMN2* who had previously been treated with either a low-dose (6•7 × 10^13^ vector genomes [vg]/kg) or therapeutic dose (2•0 × 10^14^ vg/kg) of onasemnogene abeparvovec were observed for adverse events. All ten of the children who received a therapeutic dose were alive, had maintained their previously acquired motor milestones, and did not require any new permanent ventilation support. The three children in the low-dose cohort also remained alive, with one now requiring permanent ventilation. Serious adverse events, mainly respiratory, were reported but did not result in study discontinuation or death.

The first therapy to receive approval for SMA (for types 1–3) was nusinersen, approved by the US FDA in 2016. Nusinersen, marketed as Spinraza, is an antisense oligonucleotide therapy. It modulates alternate splicing of *SMN2* such that high levels of full-length SMN are produced. Unlike Zolgensma (a one-off treatment), nusinsersen must be taken every 4 months for life and is delivered via lumbar puncture. The final efficacy and safety analysis of a phase 2, open-label, multicentre trial of nusinersen in 20 symptomatic patients was published in June in The Lancet Child & Adolescent Health. In line with previous trials of nusinersen, the treatment was found to improve attainment of developmental milestones and survival and had a favourable safety-profile. Five deaths, related to SMA progression, were reported within the 3-year follow-up period. Also in June, nusinersen was reported to be safe and well-tolerated in adult patients with ambulatory SMA. The study of the 13 adult participants, published in Frontiers of Neurology, also reported improvements in motor function and electrophysiological results over the 14-month study period. This finding is important as there have been little data regarding the effect of late SMN restoration on motor function.

Another SMN2 splicing modifier, risdiplam, is currently being tested in phase 2 and 3 clinical trials. It is derived from an earlier small molecule, RG7800, that was in development but was terminated in 2015 because of toxicity in pre-clinical studies. Risdiplam is taken as a daily, oral treatment. Early results of two trials were presented at the Cure SMA 2021 Virtual SMA Research & Clinical Care conference (virtual) in June. RAINBOWFISH (NCT03779334) is a trial of 5 pre-symptomatic babies; reportedly, 4 were able to stand unaided in the 12 months after treatment. JEWELFISH (NCT03032172), a trial for patients who have been previously treated with other SMA-targeting therapies, showed a sustained increase in SMN protein levels and maintenance of motor function. Further study will determine the long-term safety and efficacy of risdiplam, which has been made globally available in 2020 by Roche for compassionate use.

Although these therapies have been shown to alleviate the disease course for patients with SMA, poor levels of motor function have typically begun to manifest before the start of treatment and muscle tissue is already weak. For this reason, myostatin inhibitors are under investigation to increase muscle mass. Results from a phase 1 trial of apitegromab (SRK-015) were published in May in Advances in Therapy. Apitegromab is a monoclonal antibody that binds to pro- and latent forms of myostatin and blocks its activity. An increase in muscle mass and function following myostatin inhibition in post-symptomatic SMA mice was previously shown by the researchers *in* Human Molecular Genetics in 2019. The phase 1 trial reported that apitegromab was safe and well tolerated with no drug-related adverse events in healthy adult participants. A phase 2 trial (NCT03921528) of apitegromab is currently ongoing for patients with milder forms of SMA.

In recognition of the efficacy and long-term safety data of these disease-modifying treatments, some countries have begun to introduce genetic testing for SMA during newborn screening (NBS). In March, SMA Europe released their recommendation calling for all European countries to introduce NBS for SMA by 2025. According to a survey prepared by the SMA NBS World Study group, and published in June in Neuromuscular Disorders nine countries have so far opted to implement SMA screening. However, many countries highlight the shortage of cost/benefit data as a continuing barrier to implementing SMA NBS.

An Australian survey on the perspectives and psychological impact effects for parents whose children received a positive result in SMA NBS, published in EClinicalMedicine in March, reported their unanimous satisfaction with the testing programme. The parents cited an overall improvement in quality of life over time, feeling that although the result was distressing, the net benefit of the programme and support they could access as a result, outweighed this drawback.

Although SMA is still a devastating condition, recent therapeutic advances offer new hope. Early diagnosis can facilitate access to appropriate clinical care and therapies offering striking improvements to the quality of the patient's life. These successes can also inform treatments for other conditions that could benefit from similar approaches, such as gene replacement therapies for monogenic diseases and splicing modifiers for conditions including Duchenne muscular dystrophy, early-onset Parkinson's disease, and Rett syndrome.

*EBioMedicine*

